# Pathophysiology of Lung Injury Induced by Common Bile Duct Ligation in Mice

**DOI:** 10.1371/journal.pone.0094550

**Published:** 2014-04-14

**Authors:** Fumiaki Shikata, Tomohisa Sakaue, Koh-ichi Nakashiro, Mikio Okazaki, Mie Kurata, Toru Okamura, Masahiro Okura, Masahiro Ryugo, Yuki Nakamura, Takumi Yasugi, Shigeki Higashiyama, Hironori Izutani

**Affiliations:** 1 Department of Cardiovascular and Thoracic Surgery, Ehime University Graduate School of Medicine, Ehime, Japan; 2 Department of Cell Growth and Tumor Regulation, Ehime University, Proteo-Science Center, Ehime University, Ehime, Japan; 3 Department of Biochemistry and Molecular Genetics, Ehime University Graduate School of Medicine, Ehime, Japan; 4 Department of Oral and Maxillofacial Surgery, Ehime University Graduate School of Medicine, Ehime, Japan; 5 Department of Pathology, Division of Pathogenomics, Ehime University Graduate School of Medicine, Ehime, Japan; University of Pittsburgh, United States of America

## Abstract

**Background:**

Liver dysfunction and cirrhosis affect vasculature in several organ systems and cause impairment of organ functions, thereby increasing morbidity and mortality. Establishment of a mouse model of hepatopulmonary syndrome (HPS) would provide greater insights into the genetic basis of the disease. Our objectives were to establish a mouse model of lung injury after common bile duct ligation (CBDL) and to investigate pulmonary pathogenesis for application in future therapeutic approaches.

**Methods:**

Eight-week-old Balb/c mice were subjected to CBDL. Immunohistochemical analyses and real-time quantitative reverse transcriptional polymerase chain reaction were performed on pulmonary tissues. The presence of HPS markers was detected by western blot and microarray analyses.

**Results:**

We observed extensive proliferation of CD31-positive pulmonary vascular endothelial cells at 2 weeks after CBDL and identified 10 upregulated and 9 down-regulated proteins that were associated with angiogenesis. TNF-α and MMP-9 were highly expressed at 3 weeks after CBDL and were less expressed in the lungs of the control group.

**Conclusions:**

We constructed a mouse lung injury model by using CBDL. Contrary to our expectation, lung pathology in our mouse model exhibited differences from that of rat models, and the mechanisms responsible for these differences are unknown. This phenomenon may be explained by contrasting processes related to TNF induction of angiogenic signaling pathways in the inflammatory phase. Thus, we suggest that our mouse model can be applied to pulmonary pathological analyses in the inflammatory phase, i.e., to systemic inflammatory response syndrome, acute lung injury, and multiple organ dysfunction syndrome.

## Introduction

Liver dysfunction and cirrhosis affect vasculature in several organ systems, resulting in impairment of organ function that leads to increased morbidity and mortality.[1], [2] Hepatopulmonary syndrome (HPS) is a pulmonary vascular disorder associated with liver cirrhosis and is seen in 5–32% of cirrhotic patients in the setting of pulmonary microvascular dilatation-induced defects in arterial oxygenation.[1], [2] Although HPS occurs more frequently in patients with severe liver disease, patients with severe hepatic dysfunction do not always have HPS. This syndrome can also be seen in children with biliary atresia, and the same disease pattern is seen in the non-cirrhosis setting in congenital heart disease patients with a post-operative status of bidirectional cavopulmonary shunt that excludes hepatic venous return through the pulmonary vasculature.[3]–[5] Experimental rat models of HPS have enabled researchers to investigate the pathogenesis of this disease. Fallon et al. demonstrated that endothelial nitric oxide synthase (eNOS) and inducible nitric oxide synthase (iNOS) in the lungs play an important role in HPS pathogenesis.[5]–[9] Fallon et al. also reported that angiogenesis plays a role in the pathophysiology of HPS and explained the mechanisms underlying the activation of vascular endothelial growth factor A (VEGF-A), which is produced by inflammatory cells such as intravascular monocytes and induces angiogenesis.[10]

Establishment of a mouse model of HPS would help obtain greater insights into the genetic basis of the disease. Although some results have been reported for murine models of hepatic disease, mortality rates in mice are higher than those in rats, and limited data are available on lung pathology after common bile duct ligation (CBDL) in mice.[11]–[14] Our objectives were to establish a mouse model of lung injury by using CBDL and investigate its pulmonary pathogenesis for application in future therapeutic approaches.

## Materials and Methods

### Animal model

All animals were treated in accordance with standards of the Ehime University Animal Care Committee using approved animal protocols including animal ethics (Permit Number: 05R02-2). The Ehime University Animal Care Committee approved our animal protocols including animal ethics. All animals were treated in accordance with standards of the committee using the approved animal protocols. Eight-week-old Balb/c mice (n = 112) were obtained from Clea Japan Inc. (Tokyo) and were housed under barrier conditions. A standard sterilized laboratory diet and water were available ad libitum. All surgical procedures were performed utilizing clean techniques. All animals were anesthetized with ketamine (0.1 mg/g) and xylazine (0.01 mg/g) administered by intraperitoneal injection. After induction of anesthesia, a median abdominal incision was made and the common bile duct was identified. The duct was dissected carefully under a microscope and doubly ligated with 7–0 Prolene (Ethicon, Somerville, NJ) and transected. In the sham operation (control) group, the duct was dissected without CBDL. The abdominal incision was closed in two layers. After abdominal closure, 1.0 ml of 0.9% saline was injected subcutaneously into each mouse, and the mice were kept warm until recovery was confirmed. We continued to inject 1.0 ml of 0.9% saline subcutaneously every 2 d for 6 d; this procedure led to improved survival rates after surgery.

Mice were sacrificed between 1 and 4 weeks after surgery. Mice were anesthetized with ketamine (0.1 mg/g) and xylazine (0.01 mg/g). After we confirmed that mice were well sedated, they were given 50 µl of heparin (50 mg/ml) subcutaneously.[15] The trachea was surgically exposed and cannulated with a 20G Terumo Surflo Catheter (Terumo Corporation, Tokyo, Japan). Right cardiac ventricles were perfused with cold PBS (10 ml) under physiological pressure with an exit through the severed right atrium. The lungs were inflated and fixed at 23 cm pressure with 4% paraformaldehyde in PBS. The lungs, liver, heart, and spleen were carefully harvested with dissection of excess surrounding tissues.

A method for intrapulmonary shunt-fraction analysis in mice was established by Miniati et al.[15] Briefly, fluorescent microspheres (1.25×10^5^; diameter, 10 µm) (Molecular Probes, Eugene, OR) in PBS were injected into the inferior vena cava and all blood was aspirated from the transected right carotid artery. Aspirated microspheres were then counted manually under a fluorescence microscope (Olympus, Tokyo, Japan).

### Immunohistochemistry and immunofluorescence

In brief, deparaffinization and standard antigen retrieval by microwave irradiation were performed on 8-µm sections of 4% paraformaldehyde paraffin-fixed tissues. The sections were then blocked with 3% bovine serum albumin and incubated with primary antibodies against CD31 (BD Biosciences, San Diego, CA), von Willebrand factor (vWF; Chemicon International, Temecula, CA), eNOS, iNOS (BD Biosciences, San Diego, CA), Ly6G, CD68, F4/80, MMP-8, MMP-9, TIMP-1, and TIMP-4 (Abcam, Cambridge, MA). Next, secondary antibodies were applied and the samples were incubated. Sections were developed with 3,3′-diaminobenzidine (DAB; Sigma-Aldrich, St. Louis, MO) and counter-stained with hematoxylin.

Sections were immunostained using antibodies to vWF (Chemicon International, Temecula, CA) and tumor necrosis factor alpha (TNF-α; R&D Systems, Minneapolis, MN), and Alexa Fluor anti-mouse IgG for secondary fluorescent antibodies (Molecular Probes, Eugene, OR). For nuclear staining, mounting medium with DAPI (Vector Laboratories, Burlingame, CA) was used. Pictures were taken using a fluorescence microscope (Olympus, Tokyo, Japan).

Microvascular density and quantitation were assessed as previously described by Zhang et al.[10], [16] This analysis was performed in a blinded manner. Dissected lung sections that were immunostained for CD31 and vWF were used (n = 5), and pulmonary vessels with a diameter of >100 µm were excluded from the analysis. Positive-stained cells were counted at five representative locations under high magnification (40×) using ImageJ software (National Institutes of Health) and results were averaged.

### Assembly of CD31-positive cells/magnetic beads complex

Homogenized whole lungs were treated using a solution of type-1 collagenase (400 U/ml) (Worthington, Lakewood, NJ). CD31-positive cells, including pulmonary vascular endothelial cells, were collected with magnetic beads (Dynabeads; Life Technologies, Grand Island, NY) coated with anti-CD31 antibodies (BD Biosciences, San Diego, CA) as follows.[17] The cellular digest was filtered through a sterile 40-µm mesh and washed three times in 10% FBS-DMEM (Wako Pure Chemical Industries, Osaka, Japan). One milliliter of the cell suspension was placed in a tube with the magnetic beads/CD31-antibody complex and rotated for 20 min at 4°C. The bead-bound cells were then washed five times with 10% FBS-DMEM and the isolated cells were used in the following procedures.

### Real-time quantitative reverse transcriptional polymerase chain reaction (qRT-PCR)

Whole pulmonary cells and CD31-positive cells were selected as described above. Total RNA was extracted from each cell in the CBDL and sham mouse groups using Isogen II (Nippon Gene, Toyama, Japan). The RNA samples were then treated with an RNase-free DNase (Ambion, Carlsbad, CA) to remove genomic DNA contaminants. Messenger RNA was quantified using SYBR Green PCR master mix (Roche, Branford, CT) with an ABI Prism 7300 Fast Real-Time PCR system. The mRNA expression levels of endothelin-1 (ET-1), endothelial nitric oxide synthase (eNOS), endothelin type A receptor (ET-A), endothelin type B receptor (ET-B), kinase insert domain receptor (KDR), CC chemokine receptor type 1 (CCR1), chemokine CXC motif ligand 3 (CXCL3), CXC chemokine receptor 2 (CXCR2), chemokine CC motif ligand 9 (CCL9), matrix metallopeptidase 9 (MMP-9), interleukin-1 beta (IL-1b), and TNF-α were normalized to the levels of of β-actin. The primer sequences used for the amplification are shown in Table 1. The experiments were performed in triplicate and independently repeated a minimum of three times.

**Table 1:** Primer sequences for quantitative RT-PCR.

Target gene		Primer sequence 5'-3'
ET-1	forward	GTCAACACTCCCGACCACGTT
	reverse	CTGGTTTGTCTTAGGTGTTCCTC
eNOS	forward	CTGTGGTCTGGTGCTGGTC
	reverse	TGGGCAACTTGAAGAGTGTG
ET-A	forward	ACCACAGTCCATGCCATCAC
	reverse	TCAACATCTCACAAGTCATGAG
ET-B	forward	TTGGAGCTGAGATGTGTAAGC
	reverse	CAGTGAAGCCATGTTGATACC
KDR	forward	GGGATGG TCCTTGCATCAGAA
	reverse	ACTGGTAGCCACTGGTCTGGTTG
CCR1	forward	TTAGCTTCCATGCCTGCCTTATA
	reverse	TCCACTGCTTCAGGCTCTTGT
CXCL3	forward	GGAGCACCAACTGACAGGAGAGAA
	reverse	ACCACCCTGCAGGAAGTGTCAA
CXCR2	forward	GCTCTGACTACCACCCAACCTTGA
	reverse	AGAAGAGCAGCTGTGACCTGCTGT
CCL9	forward	CCAGTGGTGGGTGTACCAG
	reverse	CTCCGATCACTGGGGTTG
MMP9	forward	CGAACTTCGACACTGACAAGAAGT
	reverse	GCACGCTGGAATGATCTAAGC
IL-1b	forward	CAACCAACAAGTGATATTCTCCATG
	reverse	GATCCACACTCTCCAGCTGCA
TNF-α	forward	CTGTAGCCCACGTCGTAGC
	reverse	TTGAGATCCATGCCGTTG
β-actin	forward	CGGTTCCGATGCCCTGAGGCTCTT
	reverse	CGTCACACTTCATGATGGAATTGA

### Western blot analysis

CD31-positive cells were isolated as described above and lysed in RIPA buffer (50 mM Tris-HCl containing 0.15 m NaCl, 0.1% SDS, 1% sodium cholate, 1% Nonidet P-40, 1 mm EDTA, and 0.5 mm phenylmethylsulfonyl fluoride, pH 7.4). Proteins from lung tissues were also extracted with RIPA buffer. Equal concentrations of protein from pulmonary CD31-positive cells and whole lungs were fractionated on sodium dodecyl sulfate polyacrylamide gel electrophoresis (SDS-PAGE) and transferred to Immun-Blot PVDF Membranes (Bio-Rad Laboratories, Hercules, CA). Samples were incubated with primary antibodies against MMP-9, F4/80, CD31, eNOS, induced nitric oxide synthase (iNOS), and β-actin (BD Biosciences, San Diego, CA). The proteins were detected using an ECL Prime Western Blotting Detection System (GE Healthcare, Buckinghamshire, UK) and imaged on an LAS-4000 luminescent image analyzer (Fujifilm Life Science USA, Stamford, CT).

### Microarray analysis

CD31-positive cells were assembled from three mice in each group. Total RNA was extracted from pulmonary CD31-positive cells and whole pulmonary cells with Isogen II and collected as one sample in each group. We used 500 ng of total RNA to generate double-stranded cDNA. The cDNA was transcribed with DIG-labeled nucleotides (Roche Diagnostics, Basel, Switzerland), fragmented, and hybridized to a Gene Chip Mouse Gene 1.0 ST Array (Affymetrix, Santa Clara, CA) according to the manufacturer's instructions. These results were analyzed using the Gene Spring GX 12.1 (Agilent Technologies, Santa Clara, CA) and Ingenuity Pathway Analysis (IPA; Ingenuity Systems, Redwood, CA) software. Functional analysis by IPA identified the biological functions that were most significant to the data set. Fischer's exact test was used to calculate a p-value indicating the probability that each biological function assigned to that data set was due to chance alone. The microarray data are deposited in Gene Expression Omnibus (GEO, http://www.ncbi.nlm.nih.-gov/geo, Accession number: GSE50088) according to Minimum Information About Microarray Experiment (MIAME) guidelines.

### Protein array analysis of serum

Serum from CBDL and sham operated mice was prepared and subjected to mouse angiogenesis array (R&D Systems ARY015, Minneapolis, MN) analysis according to the manufacturer's instructions.

### Flow cytometry

Two weeks and 3 weeks after CBDL, bronchoalveolar lavage (BAL) fluid was collected from CBDL and sham operated mice to analyze the neutrophil and macrophage populations. To obtain BAL fluid, the trachea was cannulated with a 20 G angiocatheter and the lungs were lavaged five times with 1 ml of 1%FCS in PBS. The fluid was collected into one FACS tube and centrifuged at 500×g for 5 min at 4°C. The cell pellet was washed in FACS buffer, stained with CD11b, Ly6G, and F4/80 antibodies (BD Biosciences, San Diego, CA), and incubated on ice for 30 minutes. After cells were washed in FACS buffer, they were resuspended in 500 µl of FACS buffer and analyzed with FACS Calibur flowcytometer (Becton Dickinson, San Jose, CA).

### Statistical analysis

All measurement values in this study are expressed as means ± SE. All statistical analyses were performed with JMP 8.0 and analyzed using, where appropriate, either paired t test or Wilcoxon signed rank test. All tests were two sided. Results with p-values <0.05 were considered statistically significant.

## Results

**Figure 1:**
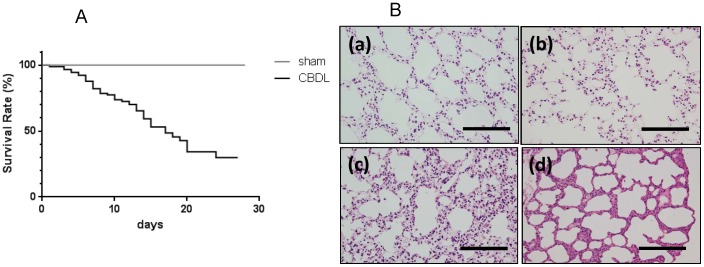
Survival curves and histological changes in common bile duct ligated (CBDL) mice and sham operated mice. (A) Kaplan-Meier survival curve in common bile duct ligated (CBDL) mice (n = 44) and sham operated (control) mice (n = 41). (B) HE-stained lung sections of sham and CBDL mice: (a) sham; (b) 1 week after CBDL; (c) 2 weeks after CBDL; (d) 3 weeks after CBDL (×20). Scale bar  = 100 µm.

**Figure 2:**
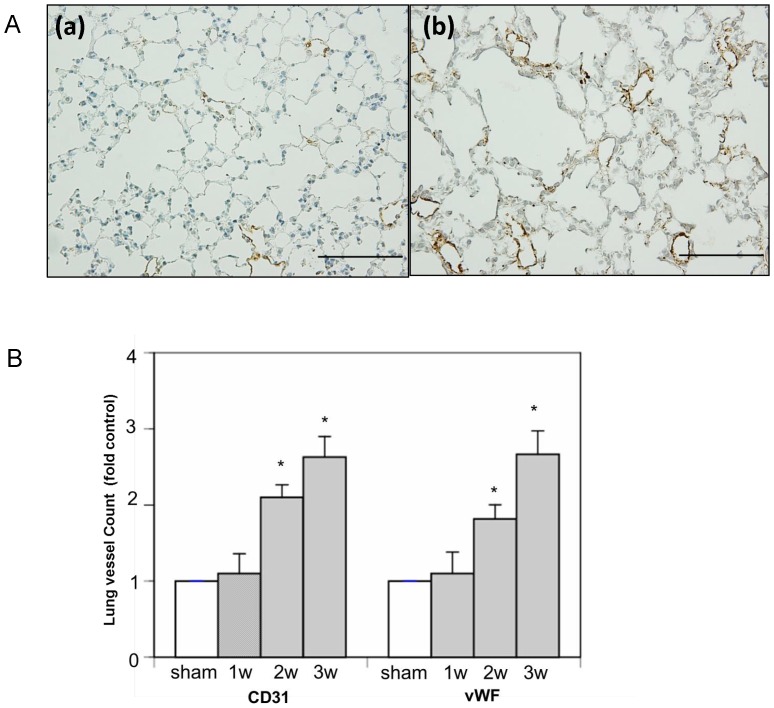
Immunohistochemical analysis of lung tissues in common bile duct ligated (CBDL) and sham-operated (control) mice. (A) Immunohistochemistry of von Willebrand factor (vWF)-stained lung section of sham and CBDL mice: (a) sham; (b) 3 weeks after CBDL. Scale bar  = 100 µm. (B) Graphical summary of lung microvessel counts by immunohistochemical analysis using CD31 and vWF antibodies in mice at 1–3 weeks after CBDL and in sham operated mice. *P<0.05 (CBDL vs. sham).

The frequency of mortality was 82.2%, 59.2%, and 34.2% at 1, 2, and 3 weeks, respectively, after CBDL (Fig. 1A). No mortality was observed in the sham group. Significant weight loss occurred beginning at 2 weeks after surgery (Table 2), and appetite loss and low activity were observed prior to death. No bile leakage was observed at autopsy in the abdominal cavity of mice that died before sacrifice. To confirm the level of liver damage resulting from CBDL surgery, serum levels of liver-function markers were measured (n = 5); all of these markers were significantly elevated from 1 week after CBDL (Table 2). The development of ascites, a parameter of liver dysfunction,[14] was observed from 1 week after surgery. To assess lung injury after surgery, arterial blood was drained from the right carotid artery for blood gas analysis. Significantly lower PaO_2_ was found in the CBDL group at 2 and 3 weeks after surgery (70.0%±2.87% and 68.4%±1.3% respectively) compared with the sham group (87.8%±1.6%) (n = 6). To confirm and compare our results with a well-established experimental hepatopulmonary syndrome model (a rat cirrhotic model of pulmonary microcirculation),[10] we applied the intrapulmonary shunt fraction calculation to our model. The resulting value for the sham group was 2.6%±1.3%. For the CBDL group, the resulting values were 4.1%±0.9% at 2 weeks after CBDL; and 4.9%±2.0% at 3 weeks after CBDL. Differences between sham and CBDL mice were not significant (P = 0.4 at 2 weeks, P = 0.3 at 3 weeks, n = 7). Histologic changes in hematoxylin-eosin staining and pulmonary vascular proliferation in immunohistochemical analysis of the lungs are shown in Figures 1B and 2A. These changes were consistent with findings of a murine CBDL model in which pulmonary angiogenic processes were confirmed by immunostaining imagery.[12]

**Table 2:** Laboratory data after common bile duct ligation in mice.

	sham		CBDL, week	
		1	2	3
AST, IU/l	64±5.0	528±72*	651±99*	647±106*
ALT, IU/l	30±2.0	520±46*	492±31*	618±107*
T. Bil, mg/dl	0.6±0.1	12±1.3*	18±2.6*	19±2.6*
ALP	315±35	1485±243*	2764±281*	2709±386*
Liver weight, g/10 g BW	0.05±0.01	0.06±0.01	0.09±0.01*	0.09±0.01*

Values given are means ± SE.

CBDL, common bile duct ligation; AST, aspartate aminotransferase; ALT, alanine aminotransferase; T. Bil, total bilirubin; ALP, alkaline phosphatase; BW, body weight. *p<0.05 vs. sham.

To investigate angiogenesis in the lung after CBDL by using methods consistent with an established rat model, we performed lung microvessel counts in which we quantified increases in microvessels at 2 and 3 weeks after CBDL in comparison with the sham group (Fig. 2A and B).[10] A significant increase in average lung microvessel count was revealed by both CD31 and vWF staining 2 weeks after CBDL, relative to the results for the sham group. Immunostaining of lungs using eNOS and iNOS antibodies, which were reported to be highly expressed after CBDL in a rat model,[6] revealed no significant differences in eNOS and iNOS expression between the CBDL and sham groups (data not shown). Elevated ET-1, ET-A, ET-B, KDR, and eNOS mRNA expression levels have also been reported after CBDL in rats.[6]–[9], [18] However, our qRT-PCR analyses revealed no significant differences in mRNA expression between the CBDL and sham groups (data not shown). We thus hypothesized that lung pathogenesis in this mouse CBDL model could be different from that in the other models, and we proceeded to apply global analysis of the gene expression profile associated with this lung pathogenesis using a microarray technique.

### Identification of genes in mouse CD31-positive pulmonary cells by microarray analysis

Based on our finding that proliferation of CD31-positive pulmonary vascular endothelial cells was more extensive after 2 weeks, we assumed that analysis of genes related to CD31-positive cells would be the key to investigating pulmonary pathogenesis after CBDL. CD31-positive pulmonary cells were selectively collected as described in the Methods, and the purity of the cell fractions was confirmed by qRT-PCR and western blot analyses.

**Figure 3:**
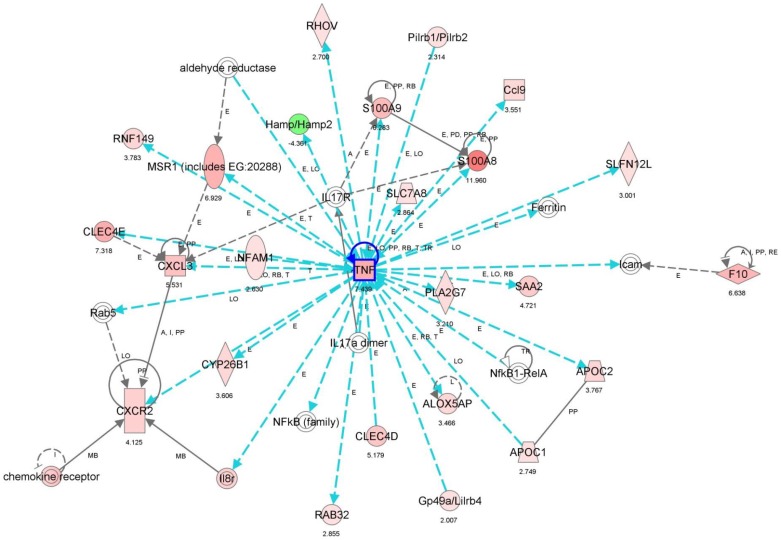
Relevance network of gene signaling pathways identified by Ingenuity Pathway Analysis. Arrow charts represent all expression values (fold changes). Color-coded gene symbols: red represents up-regulation, green represents down-regulation. Tumor necrosis factor-alpha (TNF-α) plays a central role in pulmonary pathogenesis in common bile duct ligated mice.

**Figure 4:**
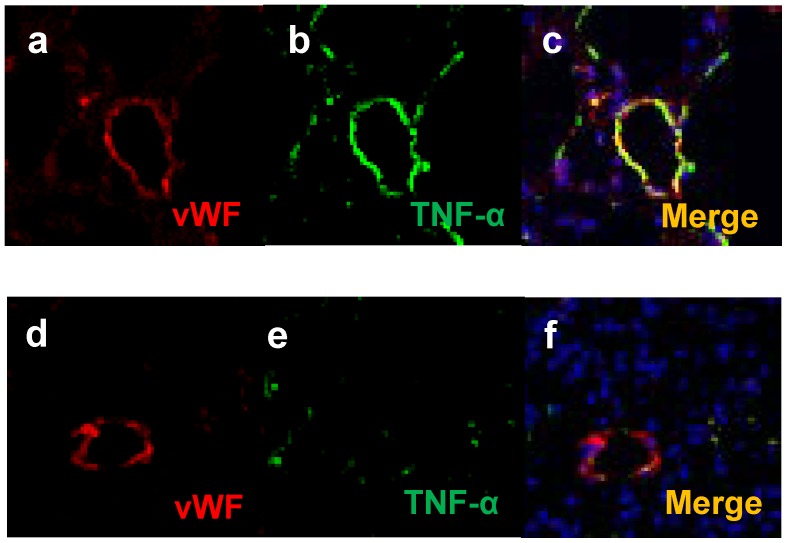
Immunofluorescent localization of von Willebrand factor (vWF) and tumor necrosis factor alpha (TNF-α). Representative images of lung tissues isolated 3 weeks after the CBDL or sham procedures showing vWF (red), TNF-α (green), and DAPI, nuclear staining marker (blue). (a–c) Staining at 3 weeks after the CBDL and (d–f) sham procedures. TNF-α was more highly expressed in pulmonary endothelial vascular cells at 3 weeks after CBDL than those of the sham operated group (original magnification, ×20).

We determined the global gene expression profiles of CD31-positive pulmonary cells in mice 2 and 3 weeks after CBDL, compared to those of sham mice, and found 79 genes that were commonly up-regulated by more than 2-fold in mice that had undergone CBDL (Table 3). This information was used to visualize the network of gene expression patterns, and 34 genes were identified as being connected directly or indirectly to TNF-α, located at the center of the network (Fig. 3). To confirm the localization of TNF-α, immunofluorescence analysis was used (Fig 4). The results indicated that TNF-α was highly expressed in pulmonary vascular endothelial cells in the CBDL mice, when compared with the sham operated mice.

**Table 3:** Genes up- or down-regulated >2.0 fold in pulmonary cells of mice at 2 and 3 weeks after CBDL, assembled by a magnetic bead/CD31-antibody complex, in comparison with levels in sham operated mice, as determined by the Gene Chip Mouse Gene 1.0 ST Array.

Gene ID	Symbol	Gene Name Fold change	2w	3w
17394	MMP8	matrix metallopeptidase 8 (neutrophil collagenase)	18.597	20.121
74145	F13A1	coagulation factor XIII, A1 polypeptide	12.429	7.512
20201	S100A8	S100 calcium binding protein A8	11.960	13.878
21926	TNF	tumor necrosis factor	7.439	2.874
56619	CLEC4E	C-type lectin domain family 4, member E	7.318	3.959
12475	CD14	CD14 molecule	7.262	4.187
13003	VCAN	versican	7.093	3.964
100034251	Gm11428	predicted gene 11428	7.012	9.548
104183|12655	Chi3l3/Chi3l4	chitinase 3-like 3	6.984	3.645
20288	MSR1	macrophage scavenger receptor 1	6.929	4.557
14058	F10	coagulation factor X	6.638	3.471
12772	CCR2	chemokine (C-C motif) receptor 2	6.389	4.536
20202	S100A9	S100 calcium binding protein A9	6.283	7.824
58217	TREM1	triggering receptor expressed on myeloid cells 1	5.96	5.005
216799	NLRP3	NLR family, pyrin domain containing 3	5.68	3.363
20310	CXCL3	chemokine (C-X-C motif) ligand 3	5.531	3.265
20716	SERPINA3	serpin peptidase inhibitor, clade A (alpha-1 antiproteinase, antitrypsin), member 3	4.557	5.632
12768	CCR1	chemokine (C-C motif) receptor 1	4.501	4.698
12489	Cd33	CD33 antigen	4.368	4.975
23845	CLEC5A	C-type lectin domain family 5, member A	4.148	4.047
12765	CXCR2	chemokine (C-X-C motif) receptor 2	4.125	5.535
100038947	Sirpb1a	signal-regulatory protein beta 1A	3.944	3.915
83382	SIGLEC8	sialic acid binding Ig-like lectin 8	3.921	3.208
12273	C5AR1	complement component 5a receptor 1	3.893	2.917
16819	LCN2	lipocalin 2	3.862	3.671
16948	LOX	lysyl oxidase	3.856	3.168
12986	CSF3R	colony stimulating factor 3 receptor (granulocyte)	3.854	4.490
16409	ITGAM	integrin, alpha M (complement component 3 receptor 3 subunit)	3.791	3.748
17395	MMP9	matrix metallopeptidase 9 (gelatinase B, 92kDa gelatinase, 92 kDa type IV collagenase)	3.576	4.147
20308	CCL9	chemokine (C-C motif) ligand 9	3.551	2.835
246256	FCGR3A	Fc fragment of IgG, low affinity IIIa, receptor (CD16a)	3.511	3.572
74442	SGMS2	sphingomyelin synthase 2	3.491	2.318
11690	ALOX5AP	arachidonate 5-lipoxygenase-activating protein	3.466	3.410
170733	Klra17	killer cell lectin-like receptor, subfamily A, member 17	3.466	3.997
20568	SLPI	secretory leukocyte peptidase inhibitor	3.433	2.644
18173	SLC11A1	solute carrier family 11 (proton-coupled divalent metal ion transporters), member 1	3.212	2.344
56221	CCL24	chemokine (C-C motif) ligand 24	3.211	2.598
27226	PLA2G7	phospholipase A2, group VII (platelet-activating factor acetylhydrolase, plasma)	3.21	3.009
18733	LILRB3	leukocyte immunoglobulin-like receptor, subfamily B (with TM and ITIM domains), member 3	3.173	3.046
13058	CYBB	cytochrome b-245, beta polypeptide	3.147	2.017
16176	IL1B	interleukin 1, beta	3.147	2.196
170743	TLR7	toll-like receptor 7	3.136	2.434
278180	VSIG4	V-set and immunoglobulin domain containing 4	3.131	9.911
12978	CSF1R	colony stimulating factor 1 receptor	3.076	2.409
19204	PTAFR	platelet-activating factor receptor	3.049	2.502
21810	TGFBI	transforming growth factor, beta-induced, 68kDa	3.004	2.992
20556	SLFN12L	schlafen family member 12-like	3.001	2.139
12182	BST1	bone marrow stromal cell antigen 1	2.925	2.327
15368	HMOX1	heme oxygenase (decycling) 1	2.844	2.563
14293	FPR1	formyl peptide receptor 1	2.748	2.280
14191	FGR	Gardner-Rasheed feline sarcoma viral (v-fgr) oncogene homolog	2.675	2.150
57248	Ly6a	lymphocyte antigen 6 complex, locus A	2.673	3.938
20351	SEMA4A	sema domain, immunoglobulin domain, transmembrane domain and short cytoplasmic domain, 4A	2.654	2.315
74039	NFAM1	NFAT activating protein with ITAM motif 1	2.63	2.750
16854	LGALS3	lectin, galactoside-binding, soluble, 3	2.627	2.037
14131	FCGR2A	Fc fragment of IgG, low affinity IIa, receptor (CD32)	2.569	2.921
12267	C3AR1	complement component 3a receptor 1	2.546	3.573
14127	FCER1G	Fc fragment of IgE, high affinity I, receptor for; gamma polypeptide	2.529	2.279
80885	HCAR2	hydroxycarboxylic acid receptor 2	2.508	2.019
380924	OLFM4	olfactomedin 4	2.497	2.717
11801	CD5L	CD5 molecule-like	2.492	5.669
12983	CSF2RB	colony stimulating factor 2 receptor, beta, low-affinity (granulocyte-macrophage)	2.444	2.262
16658	MAFB	v-maf musculoaponeurotic fibrosarcoma oncogene homolog B (avian)	2.428	2.510
22177	TYROBP	TYRO protein tyrosine kinase binding protein	2.331	2.255
11501	ADAM8	ADAM metallopeptidase domain 8	2.327	2.410
170744	TLR8	toll-like receptor 8	2.177	2.221
378460	PRAM1	PML-RARA regulated adaptor molecule 1	2.153	2.739
20375	SPI1	spleen focus forming virus (SFFV) proviral integration oncogene spi1	2.142	2.175
14728	Gp49a/Lilrb4	leukocyte immunoglobulin-like receptor, subfamily B, member 4	2.007	2.302
18507	PAX5	paired box 5	−2.068	−2.522
14128	FCER2	Fc fragment of IgE, low affinity II, receptor for (CD23)	−2.08	−3.552
12482	MS4A1	membrane-spanning 4-domains, subfamily A, member 1	−2.130	−2.302
12478	CD19	CD19 molecule	−2.183	−2.643
19695	Reg3g	regenerating islet-derived 3 gamma	−2.365	−2.158
15510	HSPD1	heat shock 60 kDa protein 1 (chaperonin)	−2.529	−2.360
12517	CD72	CD72 molecule	−2.539	−2.522
17888	MYH6	myosin, heavy chain 6, cardiac muscle, alpha	−3.71	−2.284
230899	NPPA	natriuretic peptide A	−5.536	−5.525
15505	HSPH1	heat shock 105 kDa/110 kDa protein 1	−5.641	−3.651

### qRT-PCR verification of microarray data

**Figure 5:**
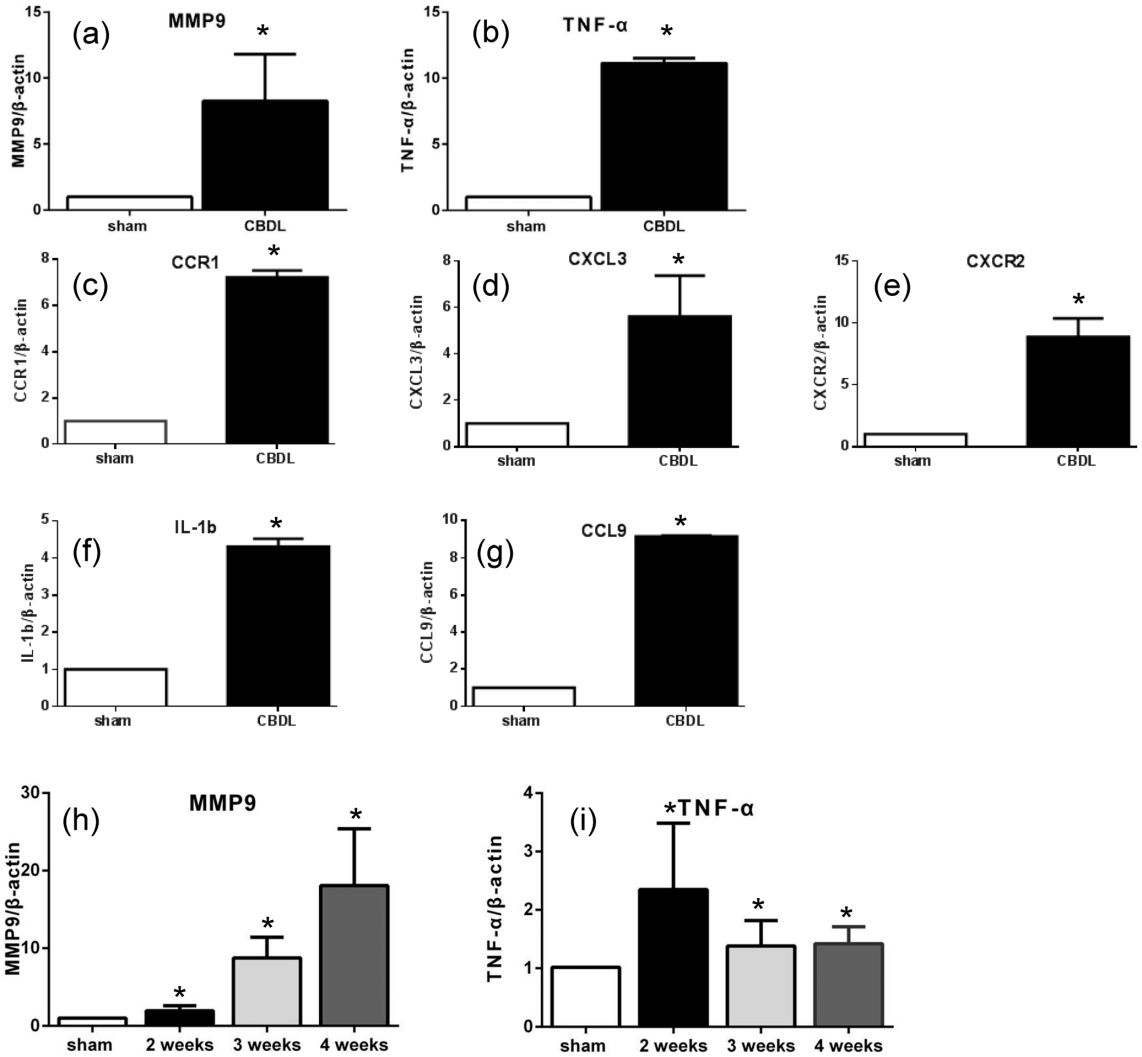
Real-time PCR analysis of representative genes identified with microarray analysis (a–g) was performed in pulmonary cells assembled by magnetic beads/CD31-antibody complexes in mice 3 weeks after CBDL (CBDL) and in sham operated controls (sham). (a) MMP9 and (b) TNF-α. (c) CCR1, (d) CXCL3, (e) CXCR2, (f) IL-1b, and (g) CCL9. (h) and (i) were performed in whole pulmonary cells of sham and 1–4 weeks after CBDL groups. (h) MMP9 and (i) TNF-α. Values are expressed as mean ± standard error (n = 3 in each group). Y-axis abbreviations: CCL9, chemokine CC motif ligand 9; MMP9, matrix metallopeptidase 9; TNF-α, tumor necrosis factor alpha; CCR1, CC chemokine type-1 receptor; CXCL3, chemokine CXC motif ligand 3; CXCR2, CXC chemokine receptor; IL-1b, interleukin-1 beta. *P<0.05 (CBDL vs. sham).

Seven genes associated with angiogenesis were selected for validation, and the directional fold change of each gene was confirmed using qRT-PCR analysis (Fig. 5a-i). Significant differences in mRNA expression of each selected gene were observed when compared with the sham operated group. Peak elevation of mRNA expression in MMP9 was observed at 4 weeks after CBDL, while that in TNF-α was observed 2 weeks after CBDL (Fig. 5h, i).

### Identification of angiogenic protein in serum by protein array analysis

**Figure 6:**
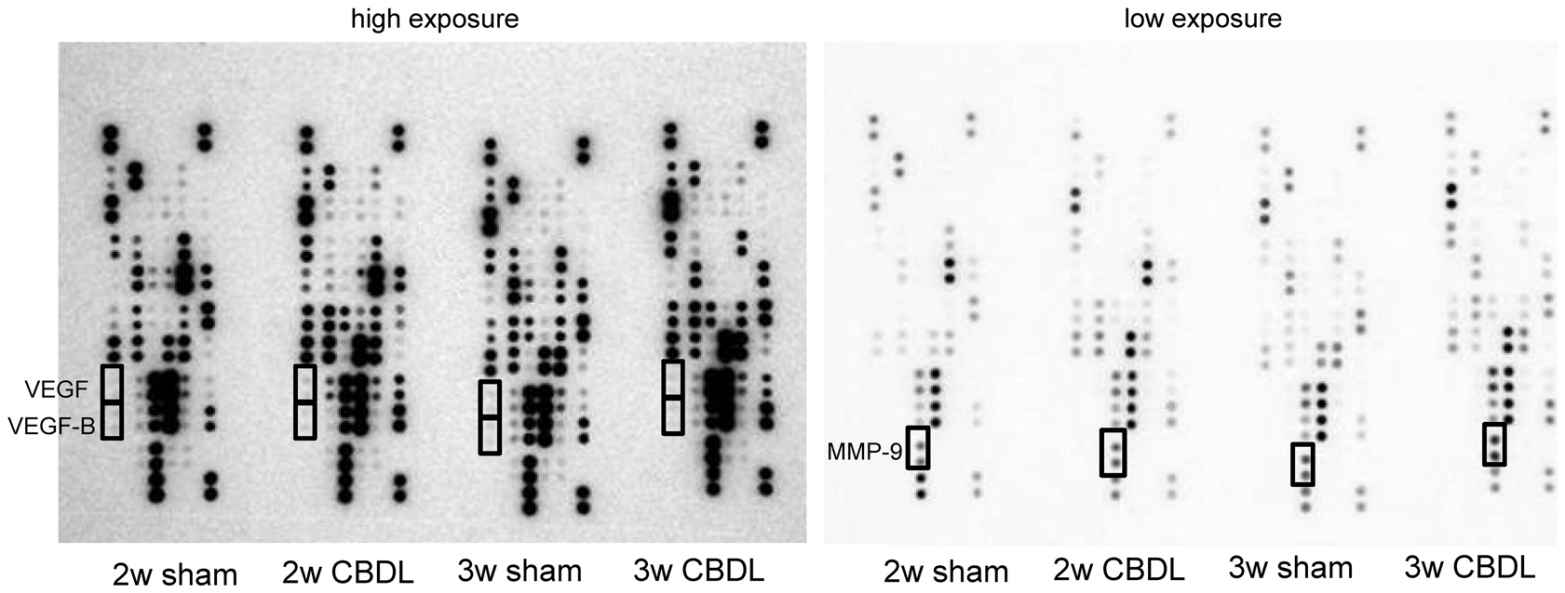
Protein array analysis of serum from common bile duct ligated (CBDL) and sham operated mice. Representative proteins identified using protein array analysis in serum isolated from CBDL mice and sham operated (sham) mice.

**Figure 7:**
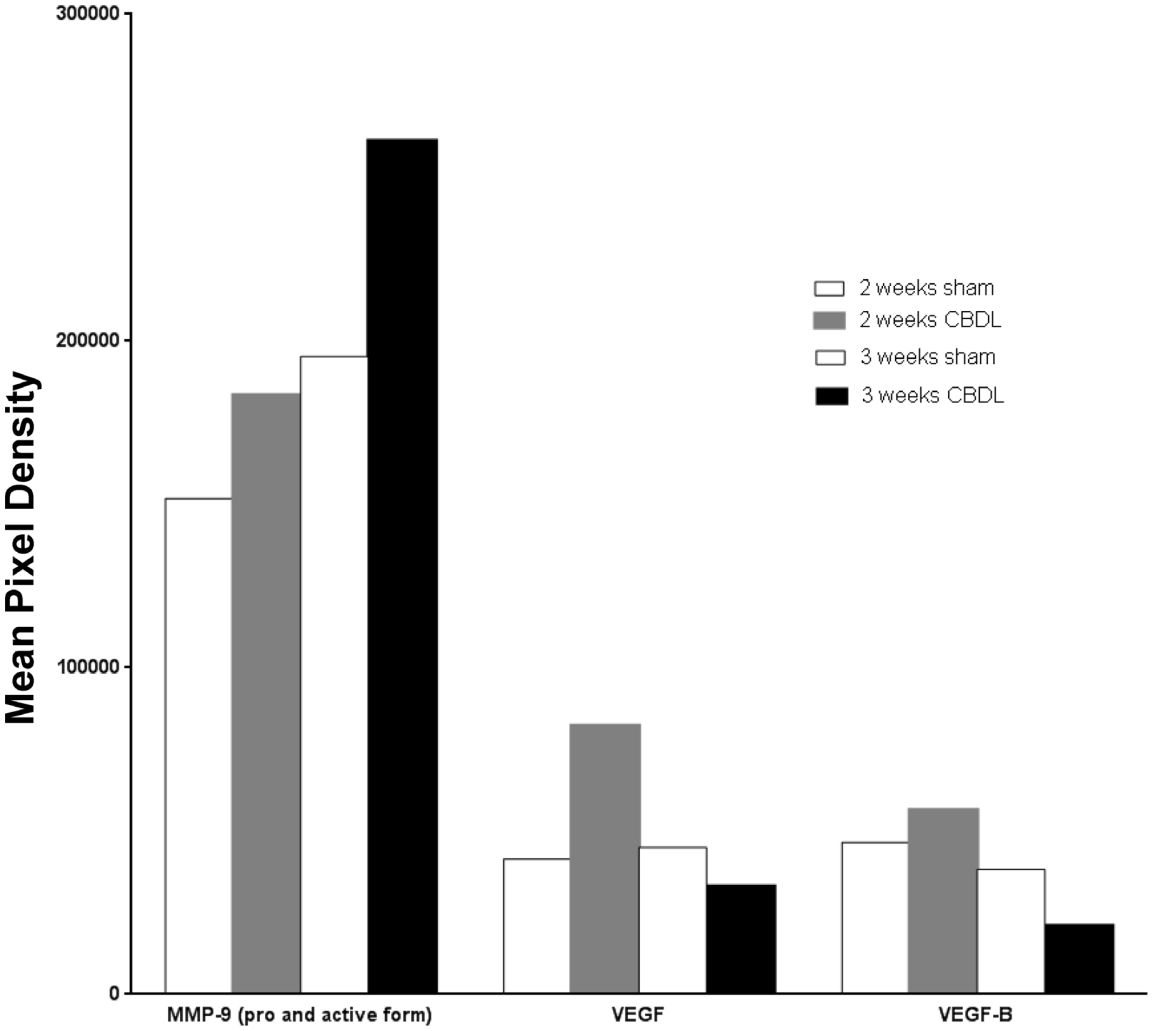
Protein array analysis of serum from common bile duct ligated (CBDL) and sham operated mice. Mean pixel density of representative proteins analyzed by protein array in serum isolated from CBDL and sham operated mice.

**Figure 8:**
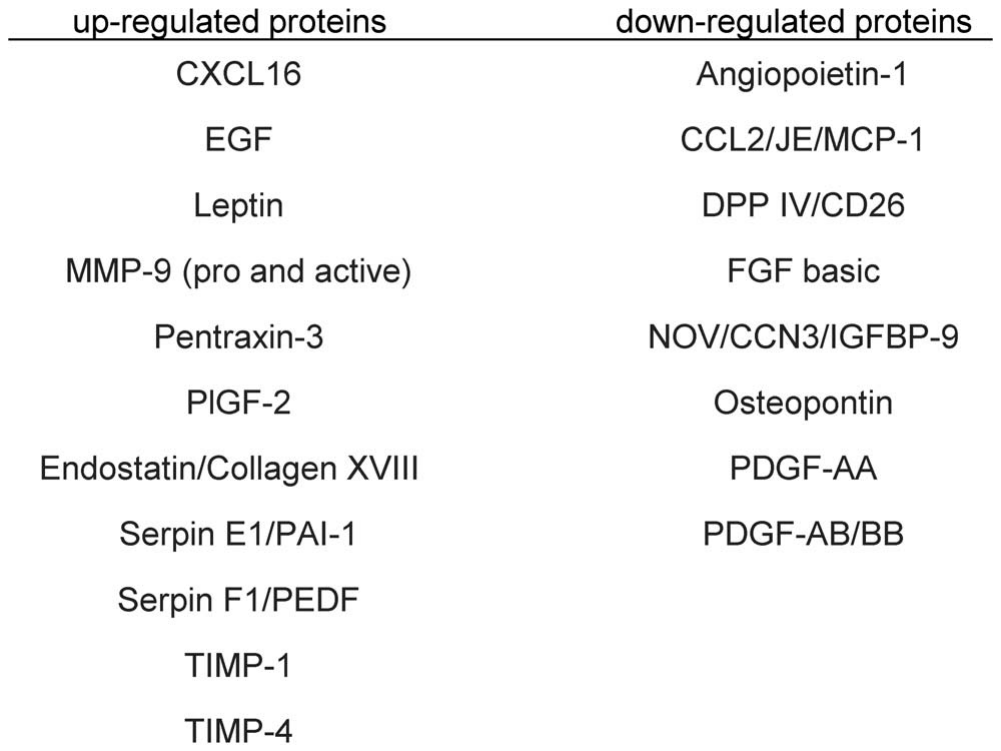
Protein array analysis of serum from common bile duct ligated (CBDL) and sham operated mice. Representative list of up-regulated and down-regulated proteins in the serum of common bile duct ligated mice, in comparison with the levels measured in sham operated mice by using protein array analysis. CXCL16, chemokine (CXC motif ligand 16); DLL4, delta-like ligand 4; DPPIV, dipeptidyl peptidase IV; EGF, epidermal growth factor; MMP-9, matrix metallopeptidase 9; FGF, fibroblast growth factor; MCP-1, monocyte chemotactic protein-1; TIMP-1, tissue inhibitor of metalloproteinase-1; CCL2/JE/MCP-1, chemokine (CC motif) ligand 2; NOV/CCN3/IGFBP-9, nephroblastoma overexpressed; VEGF, vascular endothelial growth factor; PDGF, platelet-derived growth factor.

To identify the proteins that were important to angiogenic processes, we analyzed mouse serum 2 and 3 weeks after CBDL in comparison to that of sham mice using protein array analysis focused on angiogenesis (Fig. 6 and 7, Fig. S1). This analysis indicated 10 up-regulated and 9 down-regulated proteins that were associated with angiogenesis (Fig. 8). The up-regulated expression pattern of the MMP-9 gene was also supported by protein analysis of mouse serum (Fig. 6, 7). However, no significant difference was noted between the VEGF and VEGF-B protein levels in CBDL and sham mouse serum, which was different from the data obtained in rat models (Fig. 6, 7).[10], [19]

### MMPs and TIMPs protein level and localization in impaired lungs after CBDL

**Figure 9:**
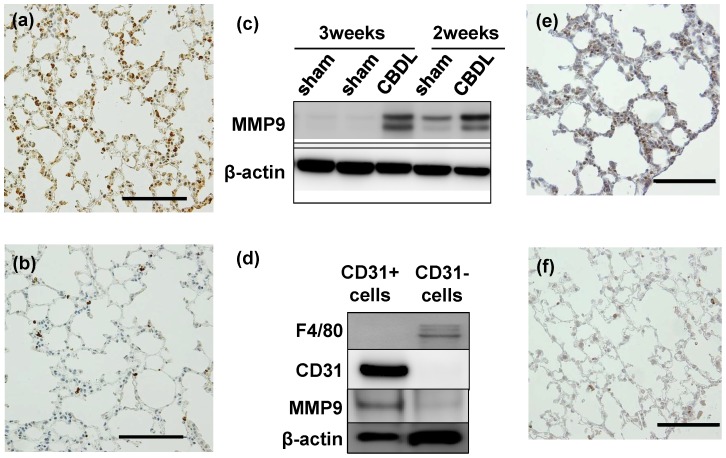
MMP8 and MMP-9 expression in mice 3 weeks after common bile duct ligation (CBDL). (a) Immunoreactivity of MMP-9 (matrix metallopeptidase) is stronger than that in sham-operated mice; (b) Immunohistochemical analysis. Scale bar  = 100 µm. (c) Immunoblotting of MMP-9 in the whole pulmonary tissue of the sham and of the 2 and 3 weeks after CBDL groups revealed that MMP-9 is highly expressed in the 2 and 3 weeks after CBDL groups, compared with the expression in the sham groups. (d) Immunoblotting of F4/80, CD31, and MMP9 in the CD31-positive pulmonary cell lysates obtained 3 weeks after CBDL showed higher levels of MMP9 expression in pulmonary vascular endothelial cells compared with those of CD31-negative pulmonary cells. (e) Immunoreactivity of MMP-8 is stronger than that in sham-operated mice; (f) Immunohistochemical analysis. Scale bar  = 100 µm.

The next step in investigating the mechanism of lung injury was to focus on MMP-9, which we expected to act as a key protein in angiogenesis after CBDL. Immunohistochemistry analyses revealed that MMP-9 was highly expressed in vascular endothelial cells in CBDL mice (3 weeks), contrary to the finding that MMP-9 was less expressed in lungs of the sham group (Fig. 9a and b). Western blot analysis of lung whole cell lysates isolated from impaired lungs also showed high expression of MMP-9 in CBDL mice compared to sham mice (Fig. 9c). In addition, CD31-positive pulmonary vascular cell lysates showed high expression of MMP9, 3 weeks after CBDL in comparison with the CD31-negative pulmonary cells. The CD31-positive pulmonary cells also had lower F4/80 expression than CD31-negative pulmonary cells (Fig. 9d). In light of the results of the microarray analysis, in which MMP8 mRNA was highly expressed in pulmonary endothelial cells, we investigated the expression of MMP8 using immunohistochemistry. We found that MMP8 was highly expressed in the lungs of the mice 3 weeks after CBDL compared with those from sham operated mice. (Fig.9e: lungs from mice 3 weeks after CBDL, Fig.9f: lungs from sham operated mice). We also examined the expression of the MMP inhibitors, TIMP-1 and TIMP-4, in lungs of CBDL and sham operated mice. A significant difference was not found among the CBDL and sham operated mice in either TIMP-1 or TIMP-4 expression in lungs. (data not shown) In addition to these data, we investigated the presence of fibrosis in lungs after CBDL using Masson trichrome staining, since we assumed that our model had the possibility of causing pulmonary fibrosis due to the high level of MMP expression. [20]–[22] However, the results showed that our model did not induce the development of pulmonary fibrosis at either 2 or 3 weeks after CBDL compared with sham operated mice (data not shown).

### Neutrophils increase in the lungs of mice after CBDL

**Figure 10:**
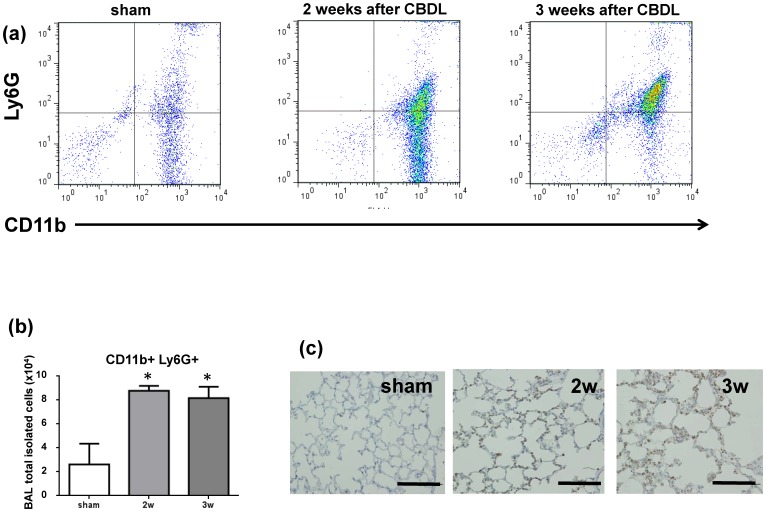
CBDL increases the number of neutrophils in bonchoalveolar lavage fluid. Neutrophils (CD11b-positive and Ly6G-positive cells) identified by flow cytometry were increased 2 and 3 weeks after CBDL. (a) Quantitation of total neutrophils in the BAL fluid showed a significant increase 2 and 3 weeks after CBDL compared with sham operated mice. (b) Ly6G expression is higher in the lungs of mice 2 and 3 weeks after CBDL compared with sham operated mice using immunohistochemical analysis. (Fig. 10c)

To investigate whether inflammatory cells contribute to the pulmonary pathogenesis in mice after CBDL, cells were collected from BAL fluid obtained from CBDL and sham operated mice and analyzed by flow cytometry. We found that CD11b positive and Ly6G positive cells, which are defined as neutrophils, were significantly increased 2 weeks after CBDL treatment. (Fig.10 a and b) Moreover, immunohistochemistry showed that Ly6G was highly expressed in lungs both at 2 and 3 weeks after CBDL, when compared with the lungs from sham operated mice. We also investigated whether the number of macrophages, i.e., those identified as CD11b-positive and F4/80-positive cells using flow cytometry analysis, were increased in the BAL fluid of mice after CBDL. Surprisingly, we found no significant increase in macrophages contrary to findings from the CBDL rat model (data not shown). [10], [19] In addition, immunohistochemistry did not show expression of either CD68 or F4/80 in the lungs of mice that had CBDL.

## Discussion

Cirrhosis results in disorders such as HPS, which is characterized by hypoxemia, high cardiac output, and extrahepatic lesions, all of which increase morbidity and mortality.[1] The same pattern of lung lesions, the pathological mechanism of which has not yet been clarified, is seen in patients with bi-directional cavopulmonary shunts.[4] Models of these diseases have been established in rats,[6], [23], [24] but to the best of our knowledge, only two studies have reported mouse models of pulmonary disease.[11], [14] We hypothesized that if a mouse model were established for this lung pathology, we could use a more extensive approach for resolving lung lesions by using transgenic or knockout mice. Therefore, we established a lung injury model by using CBDL, which incidentally had a higher survival rate than previous mouse models. [11], [14] Contrary to our expectation, lung pathology in our mouse model exhibited differences from that of rat models.[6], [10] The mechanisms responsible for the differences in pulmonary pathogenesis between mice and rats are unknown. In studies of humans with acute obstructive cholangitis (AOC) or other sepsis related to bile duct obstruction, the onset of multiple organ dysfunction (MOD) involving lungs is a complication associated with high morbidity and mortality.[1], [25] Hence, we suggest that our mouse model can be applied to pulmonary pathological analyses in the inflammatory phase, i.e., to systemic inflammatory response syndrome, acute lung injury, and MOD syndrome.[11], [26], [27]

Our findings showed that TNF-α plays an important role in development of pulmonary pathology including angiogenesis not associated with release of VEGF. On the other hand, in the rat model of CBDL, TNF-α plays an important role in pulmonary angiogenesis associated with VEGF-A-mediated angiogenic pathways.[10] This phenomenon may be explained by contrasting processes related to TNF-α induction of angiogenic signaling pathways in the inflammatory phase. TNF-α is secreted by macrophages, monocytes, and endothelial cells during inflammation. Activated neutrophils work to defend against inflammation, while overstimulated neutrophils injure tissues.[28], [29] TNF-α also induces expression of proangiogenic factors and primes vascular endothelial cells for angiogenic sprouting, while simultaneously blocking VEGF-induced proliferation in vitro.[30] TNF-α has also been reported to up-regulate VEGFR-2 expression and promote angiogenesis *in vitro*.[31], [32] Consequently, these two types of processes may help explain differences between mice and rats in angiogenic pathways after CBDL.

MMPs play an important role in the pulmonary pathogenesis of inflammatory lung diseases in human, which was reported by Kong et al.[33], [34] MMP-8 (neutrophil collagenase), which was elevated in both serum protein and pulmonary endothelial cells in this study, are released by activated neutrophils, and contributes to the formation and progression of acute lung injury and lung fibrosis.[34], [35] This pulmonary pathogenesis was confirmed by the absence of MMP-8 in mice has an antifibrotic effect to bleomycin induced lung fibrosis.[35] MMP-9 (type IV collagenase) has the ability to degrade gelatins and basement membrane collagen.[36] MMP-9 is known to exist in small quantities in healthy human lungs and to be produced at high levels by inflammatory cells (i.e., macrophages, polymorphonuclear neutrophils, and monocytes) in several lung diseases.[37] For a rodent ventilator-induced lung injury model, Kim et al. reported that lungs impaired by high tidal volume had higher expression of MMP-9 than lungs ventilated by low tidal volume and related this finding to neutrophilic inflammation.[38] Keck et al. reported that MMP-9 was a valid marker for predicting the development of lung damage and was required for migration of neutrophils into lung tissue in the rodent acute pancreatitis model.[27], [39] Johnson et al. reported that MMP-9 was required for angiogenesis in ischemic limbs and suggested that macrophages act as the source of MMP-9.[40]

In the present study, we found a high level of MMP9 production in CBDL mice serum on performing protein array analysis (Fig. 6). Strong gene expression and protein production of MMP9 were detected in CD31-positive cells (Fig. 5h). Moreover, invasive and proliferative endothelial cells were mostly observed in CBDL mice by immunohistochemical and HE staining (Fig. 1–2). Taken together, these findings suggest that MMP-9, mainly derived from endothelial cells, may play an important role in regulating angiogenesis in lung injury after CBDL. The next step will be to use MMP-9 knockout mice in analyses of treatment for cirrhosis-induced lung injury. In our model, the reason that lung fibrosis, which was assessed with Masson trichrome staining, was not strongly induced might be explained as follows; firstly, MMP-9, which is secreted from increased neutrophils during inflammation plays a role in suppression of fibrosis in the tissue.[20] Secondly, unbalanced MMP and TIMP expression during the time course we used was reported to contribute to development of experimental pulmonary fibrosis: lung MMP-2 and MMP-9 m-RNA levels have peak levels and less pulmonary fibrosis is present 1 week after induction, while MMPs inhibitors, such as TIMPs, have a different peak and are present at higher levels 3 weeks after pulmonary fibrosis induction [21], [22] Lastly, the mice strain used, which was Balb/c, might have affected the development or severity of pulmonary fibrosis, as Walkin et al. described that this strain is resistant to pulmonary fibrosis, but susceptible to hepatic fibrosis. [41]

Data from our CBDL model in mice did not mimic what is observed in rats particularly in regards to HPS pathophysiology, contrary to our expectations. The TNF-α inhibitor pentoxifylline has a beneficial effect on HPS development in rats model; however, no efficient improvement of arterial oxygenation could be observed in a pilot study of pentoxifylline administration against human HPS patients. [42] These findings suggest that HPS pathophysiology is complex and imply that a number of details regarding the mechanism of HPS are still unclear. However, we believe that our findings contribute to our understanding of HPS pathophysiology in humans and provide insights that may help in the development of efficacious therapies against this disease. [43]

In conclusion, we have shown that pulmonary pathogenesis occurs after CBDL in a mouse model and demonstrated differences between this model and other experimental animal models of hepatopulmonary syndrome. This model, which is similar to systemic inflammatory response syndrome, can be used to assess pulmonary pathology in the activated inflammation phase.

## Supporting Information

Figure S1 **List of proteins in serum of common bile duct ligated (CBDL) mice and sham operated (control) mice in protein array analysis.**
(TIF)
